# Comparative Pharmacokinetics Study of Sinomenine in Rats after Oral Administration of Sinomenine Monomer and *Sinomenium Acutum* Extract

**DOI:** 10.3390/molecules190812065

**Published:** 2014-08-12

**Authors:** Mao-Fan Zhang, Yan Zhao, Kun-Yu Jiang, Long Han, Xiao-Yue Lu, Xin Wang, Lan Zuo, Sheng-Nan Meng

**Affiliations:** Department of Pharmaceutics, School of Pharmacy, China Medical University, Shenyang 110001, Liaoning, China; E-Mails: zhangmaofan@gmail.com (M.-F.Z.); gacklen@126.com (Y.Z.); jiangkunyu87@163.com (K.-Y.J.); 15909810122@163.com (L.H.); lvxiaoyue1989@163.com (X.-Y.L.); ling007103@hotmail.com (X.W.); zuozuosix@126.com (L.Z.)

**Keywords:** pharmacokinetics, traditional Chinese medicine, sinomenine, *Sinomenium acutum*, alkaloids, HPLC

## Abstract

Various products containing sinomenine monomer and extracts of *Sinomenium acutum* have been widely applied in clinical treatments. The goal of the present study was to compare the pharmacokinetics of sinomenine in rats after oral administration of sinomenine monomer and *Sinomenium acutum* extract, and to attempt to explore potential component-component interactions between the constituents of this traditional Chinese herbal medicine. A reliable and specific reversed phase high performance liquid chromatography method was developed to analyze sinomenine in rat plasma. Pharmacokinetic parameters for sinomenine were processed by non-compartmental analysis. The results showed that the maximum concentration, the area under the concentration-time curve, clearance and the apparent volume of distribution of sinomenine in the *Sinomenium acutum* extract statistically differed from those of sinomenine monomer (*p* < 0.05); however, the mean residence time, time of peak concentration, and half-life did not show significant differences between the two groups. These findings suggested that some additional components in the *Sinomenium acutum* extract may decrease the absorption of sinomenine. The complex interactions between sinomenine and other components of the herbal extract could result in the altered pharmacokinetic behavior of sinomenine, which may subsequently cause different therapeutic and detoxification effects.

## 1. Introduction

Traditional Chinese medicine (TCM) originated in ancient China and has evolved over thousands of years. Nowadays, TCM encompasses many different practices based on the guidance of the theory of traditional Chinese medical science and has been extensively used side by side with Western medicine in most hospitals and clinics in China [1,2,3,4]. *Sinomenium acutum* is a deciduous twining vine belonging to the Menispermaceae family plant, which is one of the most important and highly valued plant families in TCM for the treatment of rheumatalgia, rheumatism, and arthralgia [5]. *Sinomenium acutum* contains many alkaloids with various skeletal structures and their biological activities have been widely studied [6,7,8,9].

**Figure 1:**
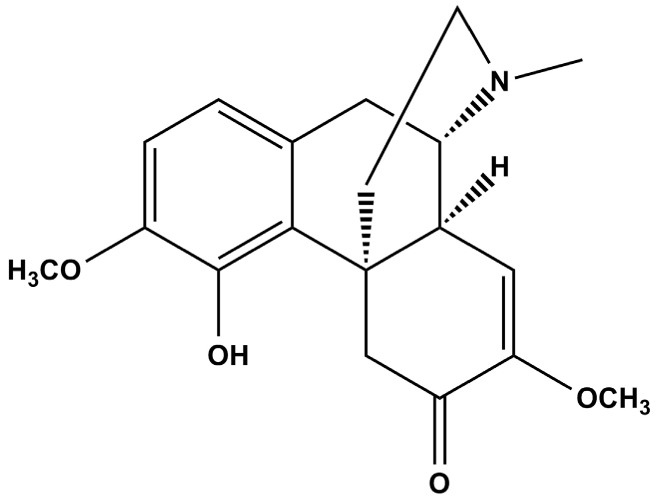
Chemical structure of sinomenine.

Sinomenine (7,8-didehydro-4-hydroxy-3,7-dimethoxy-17-methylmorphinane-6-one, Figure 1) is the main biomonomer alkaloid that is typically isolated from the Chinese medicinal plant *Sinomenium acutum*, Rehder & E.H. Wilson. Previous pharmacological studies have indicated that sinomenine has comprehensive biological activities, including immunosuppression [10], an anti-arthritic effect [11], anti-inflammatory properties [12], inhibition of lymphocyte proliferation [13], the prevention of antagonizes cartilage degradation and chondrocyte apoptosis [14]. It is used as an immunosuppressive drug in the treatment of rheumatic and arthritic diseases. Up to now, a variety of drug products from sinomenine monomer, such as enteric-coated sinomenine hydrochloride tablets and ZhengqingFengtongning injections, and herbal medicines from *Sinomenium acutum*, for example, ZhengqingFengtongning tablets and sustained-release tablets, *etc.*, have been applied in clinical treatments.

The pharmacokinetic study of TCMs is important and necessary because studies involving laboratory animals may give useful indications for TCM research and development, and it also supports the study of preclinical toxicology in animals, allowing the toxicity data of TCMs to be extrapolated to humans. Knowledge of the kinetics and effects (pharmacodynamics) of drugs in animals is necessary for therapy in humans [15,16]. However, the complexity and underlying conceptual foundations of TCM force researchers to seek evidence of whether and how it works through, for example, pharmacokinetic studies [17]. Many factors could restrict the progress of pharmacokinetic research, namely, the complexity of the medicinal components, multi-target effects, and imperfect evaluation methods, *etc*. The complexity of Chinese medicine [18,19,20] results, in part, from the fact that even a single Chinese herb contains a multitude of compounds, as well as the key component(s) (the effective monomer(s)). Each of these compounds may interact and pharmacokinetic studies [21,22,23] focused on investigating the possible pharmacokinetic differences between the compounds are vital, in particular, those that explore herb-herb or component-component interactions following oral administration of the monomer *versus* the herbal extract.

Previously, several pharmacokinetic studies of sinomenine have been carried out in rats [24,25], beagle dogs [26], and humans [27]. However, studies regarding sinomenine as the main component from *Sinomenium acutum* extracts are still lacking. An ever increasing number of studies [28,29,30] have indicated that the pharmacokinetics and therapeutic effects of the monomer in its pure form and as part of the herbal extract are different. In some situations these differences have been shown to be extensive and may lead to multi-component synergistic or inhibitory interactions. Currently, there are a large number of sinomenine products and *Sinomenium acutum* extracts, which contain sinomenine as the bioactive ingredient, and these products have been applied in clinical practice. Information regarding the differences in the pharmacokinetics of the pure monomer and sinomenine constituents in extracts of *Sinomenium acutum* would enhance our understanding and contribute to the safety and efficacy of sinomenine in clinical applications. The goal of the present research is to study and compare the pharmacokinetic profiles of pure sinomenine and sinomenine in a *Sinomenium acutum* extract when administered at approximately the same doses to rats via the oral route. Furthermore, we explore the interactions between sinomenine and the other components of *Sinomenium acutum* extract.

## 2. Results and Discussion

### 2.1. Chromatographic Separations

**Figure 2:**
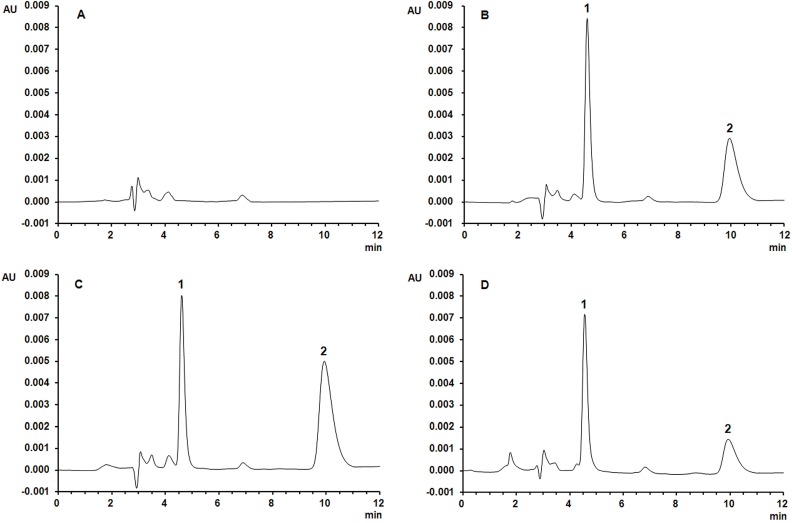
Typical chromatograms: (**A**) blank rat plasma; (**B**) blank rat plasma spiked with sinomenine and IS; (**C**) rat plasma sample after oral administration of sinomenine monomer; (**D**) rat plasma sample after oral administration of *Sinomenium acutum* extract. Peak 1: IS; peak 2: sinomenine.

A specific and reliable method to determine the concentration of sinomenine in rat plasma has been established in this study. A mixture of methanol and 30 mmol/L KH_2_PO_4_ (40:60, v/v) was found to be the most suitable medium to separate sinomenine and theophylline (internal standard, IS), with retention times of 10.6 and 5.1 min, respectively. The position of sinomenine was confirmed using the independently generated standards. Sinomenine and theophylline were eluted without any endogenous interference from the blank rat plasma (Figure 2).

### 2.2. Linearity and Sensitivity

The method validation was conducted with blank rat blood. The standard curves were linear in the concentration range of 0.2–50.0 μg/mL for sinomenine and could be described by the regression equation: *Y* = 0.148*X* − 0.007 (*R*^2^ = 0.9985). The lower limits of quantitation (LLOQ) was 0.2 μg/mL, which served as the lowest concentration on the standard curve.

### 2.3. Precision and Accuracy

The assay precision and accuracy results are shown in Table 1. The intra- and inter-day precisions and accuracy were determined by replicate analyses of the Quality control (QC) samples (*n* = 5) at three levels of concentrations (low, middle, and high). These results demonstrate that the precision and accuracy values are well within the 15% acceptance range.

**Table 1:** Extraction recovery, matrix effect, intra-day and inter-day precision and accuracy data from HPLC analysis of sinomenine.

Analyte	Concentration	Extraction Recovery (*n* = 3)	Matrix Effect (*n* = 3)	Intra-Day (*n* = 5)		Inter-Day (*n* = 5)		Accuracy (*n* = 5)
	(μg/mL)	Mean ± SD (%)	Mean ± SD (%)	Mean ± SD (μg/mL)	RSD (%)	Mean ± SD (μg/mL)	RSD (%)	RE (%)
Sinomenine	0.25	79.3 ± 3.4	89.8 ± 4.1	0.268 ± 0.029	10.9	0.287 ± 0.025	8.6	7.2
	4.00	78.5 ± 9.8	91.2 ± 2.8	4.16 ± 0.18	4.3	4.20 ± 0.23	5.5	3.3
	40.0	83.4 ± 4.2	93.5 ± 3.7	42.9 ± 1.9	4.4	41.9 ± 2.91	6.9	7.2

### 2.4. Recovery, Matrix Effect and Stability

The mean extraction recoveries determined using three QC samples replicate at three concentration levels (the same concentrations as the QC samples) in rat blood are shown in Table 1. Methanol and dichloromethane were used to extract sinomenine in the blood samples as results show it has the highest extraction recovery. With respect to the matrix effect, all the results were in the range of 85%–115%. It suggested that there was no measurable matrix effect that interfered with sinomenine determination in the rat blood.

The stability of sinomenine in rat blood was evaluated by analyzing three replicates of the QC samples at three different concentrations after short-term (25 °C, 4 h), long-term cold storage (−20 °C, 14 days), post processing (20 °C, 24 h), and after three freeze-thaw cycles (−20 to 25 °C). The RSDs for sinomenine were found to be in the range of 4.5%–11.2%. All the above results showed that a reliable, reproducible, and robust method for the analysis of sinomenine in rat blood samples has been developed and validated.

### 2.5. Pharmacokinetic Studies

**Figure 3:**
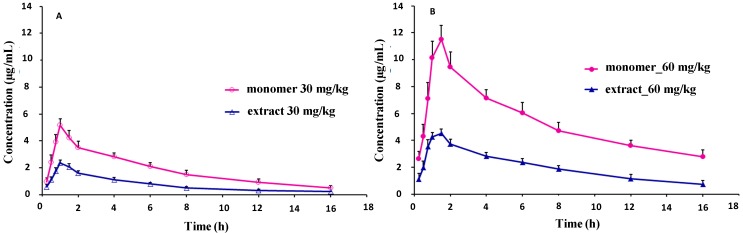
Representative curves of sinomenine concentrations *versus* time profiles in rat plasma after oral administration of sinomenine monomer (○,●), and the *Sinomenium acutum* extract (△,▲). (**A**) plot for 30 mg/kg sinomenine monomer and *Sinomenium acutum* extract; (**B**) plot for 60 mg/kg sinomenine monomer and *Sinomenium acutum* extract.

The validated analytical method was employed to study the pharmacokinetic behavior of sinomenine in rats after oral administration of the pure monomer and *Sinomenium acutum* extract, which were given at the same equivalent doses (30 and 60 mg/kg sinomenine). The mean plasma concentration-time curves of sinomenine and the herbal extract after oral administration at different doses are presented in Figure 3. The pharmacokinetic parameters of sinomenine monomer and *Sinomenium acutum* extract were calculated by the PK analysis software DAS 2.1.1 with the non-compartmental method. The fitted pharmacokinetic parameters are shown in Table 2.

**Table 2:** Pharmacokinetic parameters of sinomenine in rat plasma after oral administration of sinomenine and * Sinomenium acutum* extract (30 and 60 mg/kg) (*n* = 6).

Parameters	Sinomenine		*Sinomenium acutum* extract	
	30 mg/kg	60 mg/kg	30 mg/kg	60 mg/kg
*T*_max_ (h)	1.083 ± 0.204	1.500 ± 0.316	1.083 ± 0.204	1.333 ± 0.258
*C*_max_ (μg/mL)	5.235 ± 0.390	11.581 ± 0.942	2.397 ± 0.203 **	4.650 ± 0.186 **
AUC_0- *t*_ (mg·h/L)	29.206 ± 4.062	78.879 ± 5.129	11.824 ± 0.690 **	32.205 ± 2.723 **
AUC_0–∞_ (mg·h/L)	32.823 ± 5.368	94.024 ± 11.248	13.310 ± 1.136 **	39.966 ± 8.740 **
*t*_1/2z_ (h)	4.875 ± 0.635	5.767 ± 1.590	4.997 ± 1.314	6.442 ± 2.675
*CL*_z_/F (L/kg/h)	0.935 ± 0.152	0.646 ± 0.077	2.268 ± 0.195 **	1.553 ± 0.289 **
*V*_z_/F (L/h)	6.491 ± 0.704	5.256 ± 1.021	16.145 ± 3.515 **	13.594 ± 2.458 **
MRT_inf_ (h)	7.304 ± 0.889	8.801 ± 1.914	7.138 ± 1.416	9.582 ± 3.480

Values are mean ± SD; * *p* < 0.05, ** *p* < 0.01 compared with the level of sinomenine at the same dose.

As can be seen in Table 2, after the oral administration of doses of 30 and 60 mg/kg, the sinomenine monomer was absorbed and reached a maximum concentration (*C*_max_) of (5.235 ± 0.390) μg/mL and (11.581 ± 0.942) μg/mL, respectively. Meanwhile, the *C*_max_ of sinomenine from the herbal extract at the same doses were (2.397 ± 0.203) μg/mL and (4.650 ± 0.186) μg/mL, respectively. The area under curve of the concentration-time profile (*AUC*_0-*t*_) of sinomenine was (29.206 ± 4.062) mg·h/L at a dose of 30 mg/kg, which was lower than its *AUC*_0-*t*_ (78.879 ± 5.129 mg·h/L) following a dose of 60 mg/kg. The *AUC*_0-*t*_ of sinomenine in the *Sinomenium acutum* extract was (11.824 ± 0.690) and (32.205 ± 2.723) mg·h/L at doses of 30 and 60 mg/kg, respectively. These results indicated that the values of *C*_max_ and *AUC*_0-*t*_ of sinomenine from the crude extract were lower than those of the pure sinomenine monomer, especially at the higher dosage. However, on the contrary, for clearance (*CL*_z_/*F*) and the apparent volume of distribution (*V*_z_/*F*), the parameters from the herbal extract were higher than those of the pure sinomenine monomer at the same dosage. Most pharmacokinetic parameters, such as *C*_max_, *AUC*_0-*t*_, *CL*_z_/*F* and *V*_z_/*F*, of sinomenine in the *Sinomenium acutum* extract statistically differed from those of the pure monomer (*p* < 0.05); however, the mean residence time (*MRT*_inf_), the time of peak concentration (*T*_max_), the and half-life (*t*_1/2z_) did not show significant differences between the two groups.

Examining the results, it can be found that there were two factors involved in the pharmacokinetics of sinomenine monomer and sinomenine from the crude herbal extract. The first one is the additional components present in the *Sinomenium acutum* extract, which may influence the actions of sinomenine *in vivo*. For example, the results showed that the *MRT* is almost the same for the two preparations but *AUC* for the sinomenine in the herbal extract were much lower than those of sinomenine monomer, the difference in *C*_max_ which is mainly attributed to the fact. The differences revealed that the complex constituents in the crude extract may suppress the absorption of sinomenine or complete the transmembrane transport of sinomenine, which is consistent with experiments on the absorption behavior of sinomenine monomer and sinomenine from the crude extract using a rat *in situ* perfusion model (data not shown). In addition, the affinity of the compound from extract may be much better than sinomenine, and sinomenine could not play its P-glycoprotein (P-gp) inhibitory effect even though it is an inhibitor of P-gp [31,32]. By our results, *CL_z_*/*F* increases significantly with the extract. In general, these increases may come from *CL_z_* and *F*. But for our result, we think the increases are mainly caused by *F*. Because if the clearance (*CL_z_*) increases, the *MRT* and *t_1/2z_* will decrease accordingly. While for our results, the parameters of *MRT* and *t_1/2z_* have not significant differences, this means that *CL_z_* does not make *CL_z_*/*F* changed. On the other hand, *F* is a parameter involved with the rate and extent of compound absorption, by our data, the *T_max_* of the two preparations is similar, but the *C*_max_ or the *AUC* of the extract is indeed much lower than that of sinomenine monomer, the absorption of sinomenine in the herbal extract is slower than that of sinomenine monomer, so the *F* of sinomenine is reduced when it is administered as an herbal extract, and makes the *CL_z_*/*F* with the extract increases significantly.

Another factor was also noted to influence the pharmaceutical actions of sinomenine in our preparations. *C*_max_, *AUC*_0-*t*_, *CL*_z_/*F*, and *V*_z_/*F* changed with the dosage. The values of *C*_max_ and *AUC*_0-*t*_ increased with the dosage; however, *CL*_z_/*F* and *V*_z_/*F* decreased with an increasing dosage. These results indicate that the absorption of sinomenine is additive, and the elimination of sinomenine reduces with ascending dosages of sinomenine from 30 to 60 mg/kg, which may lead to cumulative effects. This is supported by our finding that the duration of elimination was extended at higher doses of sinomenine. As such, cares should be taken with regards to the interactions and toxicity of sinomenine when *Sinomenium acutum* extract products are used in clinical treatments.

For our data, the values of pharmacokinetic parameters compared to others [24,32,33,34,35] are quite different. The main reasons may be caused by the different protocols used in different paper, such as the different route of administration for sinomenine, the different amount and form of sinomenine, the different species of rats, and so on. Furthermore, if sinomenine was determined as a target constituent of compound preparations, other components in the preparations could influence the pharmacokinetic action of sinomenine.

The bioactive compounds in plants may play various kinds of effects, and it is necessary to identify or determine what kind and amount of the components were contained in the extract, further elucidate the facts [36,37,38]. So far, there are a lot of bioactive compounds besides sinominine in *Sinomenium acutum* extract were identified, such as sinoacutine, acutumin, N-acutumidine, sinactine, isosinomenine, stigmasterol, β-sitosterol, syringaresinol, *etc.* Additional studies would be needed to determine and analyze the bioactive effects or influences of the bioactive compounds in the extract.

As noted earlier, traditional Chinese medicines are complex systems, and even with single herbal preparations the pharmacokinetics can be complicated by the combined effects arising from the multiple components in the herbal extract, thereby influencing the effects and/or the detoxifications of the active ingredient(s). Future studies need identifying which components may affect or interact with sinomenine.

## 3. Experimental

### 3.1. Animals

Male Wistar rats, weighing 180–220 g, were provided by the Laboratory Animal Center of China Medical University (Shenyang, China) and were kept in an environmentally controlled room (temperature 25 ± 2 °C, humidity 50% ± 5%, and 12 h dark/light cycle) for at least 1 week before the experiments. The rats were fasted overnight with free access to water before the date of the experiment. The use of animals in the present study was permitted by the Ethics Committee of China Medical University and all animal studies were carried out according to the Guide for Care and Use of Laboratory Animals.

### 3.2. Materials and Reagent

*Sinamenium acutum* (Thunb.) Rehd. et Wils. was purchased from Hebei Anguo Shenhe Traditional Chinese Medicine Co., Ltd (Anguo, China) and was authenticated by Zai-Xing Chen (Pharmaceutical Central Laboratory, China Medical University, Shenyang, China). Sinomenine (98.04% purity) was purchased from Xi’an XiaoCao Botanical Development Co., Ltd (Xi’an, China). Theophylline (IS) was bought from the National Institute for the Control of Pharmaceutical and Biological Products (Beijing, China). Methanol (HPLC grade) was obtained from Honeywell (Muskegon, MI, USA). Deionized water was prepared from a Millipore (Billerica, MA, USA) water purification system and was filtered through a 0.22 µm membrane (Millipore) prior to use. All other reagents were of analytical grade.

### 3.3. Apparatus and Chromatographic Conditions

For our sample analysis, high-performance liquid chromatography (HPLC) was performed using a Waters Alliance HPLC 2695 series (Waters, Manchester, UK) with separation on a reversed-phase C_18_ Dikma Diamonsil^TM^ analytical column (200 mm × 4.6 mm, 5 µm). The mobile phase consisted of methanol and 30 mmol/L KH_2_PO_4_ (40:60, v/v) at a flow rate of 1.0 mL/min and the column temperature was maintained at 35 °C. The detection wavelength was set at 263 nm for acquiring chromatograms. The injection volume was 50 µL.

### 3.4. Preparation of Sinomenium acutum Extract

Dry slices of *Sinomenium acutum* were crushed to a 20 mesh pulverization degree. The pulverization powder was initially soaked for 2 h with eight times the volume of 70% (v/v) ethanol and was then extracted three times. During the extraction process the extraction fluid was collected, and then condensed using a rotary evaporator. The yield of the extract was approximately 14.46% (w/w). The concentration of sinomenine in the extract was measured by HPLC. The concentrated extract was stored at 4 °C until required.

### 3.5. Animal Experimental Design

Rats were randomly divided into four groups (*n* = 6). Group I and II were administrated an oral dose of 30 and 60 mg/kg sinomenine, respectively. Group III and IV were administrated an oral dose of *Sinomenium acutum* extract, which was equivalent to 30 and 60 mg/kg sinomenine, respectively. Both the sinomenine monomer and the extract were suspended in 0.5% sodium carboxymethyl cellulose (CMC-Na) aqueous solution just before use. 

Blood samples of approximately 0.3 mL were collected in heparinized Eppendorf (EP) tubes via the retro-orbital sinus of rats at 0, 0.25, 0.5, 0.75, 1, 1.5, 2, 4, 6, 8, 12, and 24 h after drug administration, and immediately separated by centrifugation at 5,000 rpm for 10 min. The plasma was then collected and frozen at −40 °C until analysis.

### 3.6. Plasma Sample Preparation

To determine the concentration of sinomenine in the plasma, frozen plasma samples were thawed at room temperature and vortex-mixed to achieve thorough mixing prior to precipitation. Then, to 100 µL aliquots of plasma in 1.5 mL EP tubes, 50 µL theophylline (5.0 μg/mL) and 50 µL methanol were added and mixed well. Then, 1.5 mL dichloromethane was added and vortex-mixed for 3 min. After centrifugation at 10,000 rpm for 10 min, the supernatant was transferred into a 1.5 mL EP tube and evaporated to dryness under a stream of nitrogen gas at 45 °C. The residue was reconstituted in 100 µL of the mobile phase (methanol and 30 mmol/L KH_2_PO_4_, 40:60, v/v) by vortex-mixing for 30 s and centrifuged at 14,500 rpm for 10 min. The supernatant (50 µL) was collected and injected into the HPLC system for analysis.

### 3.7. Preparation of Calibration Standards and QC Samples

A stock solution of sinomenine was prepared in methanol at a concentration of 0.2 mg/mL and a stock solution of the IS was prepared by dissolving theophylline in methanol to a final concentration of 5.0 μg/mL. Both stock solutions were stored at 4 °C until use.

The calibration standard samples were freshly prepared by spiking blank plasma with the appropriate working solution, yielding concentrations of 0.2, 0.5, 1.0, 5.0, 10.0, 20.0, and 50.0 μg/mL sinomenine. The samples were then processed as described in the plasma sample preparation section. QC samples, which were used for intra- and inter-day accuracy and precision, extraction recovery, and stability studies, were prepared in the same way as the calibration standard samples at concentrations of 0.25, 4.00, and 40.0 μg/mL sinomenine.

### 3.8. Method Validation

The specificity and selectivity of the method was evaluated by comparing the chromatograms of blank plasma, blank plasma spiked with IS/analytes and rat plasma samples. A calibration curve was constructed based on the peak-area ratios of the analyte to the IS (*Y*) *vs.* the concentrations of the spiked analyte(*X*) using a linear least squares regression model (1/*X*^2^ weighting). The LLOQ served as the lowest concentrations on the standard curve. Intra- and inter-day precisions were determined by assessing the measured results of the QC samples at low, medium, and high concentrations. Precision was evaluated as the relative standard deviation (RSD), while accuracy (%) was evaluated by the percentage difference between the mean measured concentrations and the spiked concentrations, expressed as the relative error (RE). Extraction recoveries were determined by comparing the ratio of analyte peak areas of the extracted QC samples with those of un-extracted standard solutions at the same nominal concentrations. The matrix effects were measured by comparing the peak areas of blank plasma extracts spikes with analytes with those of pure standard solution containing equivalent amounts of the analytes. Stability was checked by comparing the measured results with those of the freshly prepared samples of the same concentration under different storage conditions: short-term stability at 25 °C for 4 h; long-term stability at −20 °C for 14 days; post-preparative stability at room temperature for 24 h; after three freeze-thaw cycles.

### 3.9. Pharmacokinetic and Data Analysis

Pharmacokinetic parameters for two kinds of sinomenine were processed by non-compartmental analysis using PK analysis software DAS 2.1.1 (Mathematical Pharmacology Professional Committee of China, Shanghai, China). Data are presented as mean ± SD. Comparisons of the pharmacokinetic data were performed by Student’s t-test and the statistically significant difference was set at a value of *p* < 0.05 (SPSS statistical software package, version 17.0, SPSS Inc., Chicago, IL, USA).

## 4. Conclusions

The present study showed that the pharmacokinetic profiles of pure sinomenine monomer and sinomenine from a *Sinomenium acutum* extract greatly varied after oral administration in rats. Some components in the *Sinomenium acutum* extract may decrease the absorption of sinomenine, which may account for the lower blood concentration of sinomenine after administration of the herbal extract. The complex interactions between sinomenine and other components of the herbal extract could result in the different pharmacokinetic behavior of sinomenine, which may cause subsequently result in unexpected clinically therapeutic and detoxification effects. Since various sinomenine products are widely used in clinical practice, our initial findings of the comparative pharmacokinetics for the pure monomer and herbal extract contribute to the efficacy and safety of sinomenine and the *Sinomenium acutum* extract. Furthermore, our results may enrich the rational for different sinomenine products and their clinical applications.
